# Alpha-Synuclein Gene Alterations Modulate Tyrosine Hydroxylase in Human iPSC-Derived Neurons in a Parkinson’s Disease Animal Model

**DOI:** 10.3390/life14060728

**Published:** 2024-06-05

**Authors:** Luis Daniel Bernal-Conde, Verónica Peña-Martínez, C. Alejandra Morato-Torres, Rodrigo Ramos-Acevedo, Óscar Arias-Carrión, Francisco J. Padilla-Godínez, Alexa Delgado-González, Marcela Palomero-Rivero, Omar Collazo-Navarrete, Luis O. Soto-Rojas, Margarita Gómez-Chavarín, Birgitt Schüle, Magdalena Guerra-Crespo

**Affiliations:** 1 Laboratory of Regenerative Medicine, Physiology Department, Faculty of Medicine, National Autonomous University of Mexico, Mexico City 04510, Mexico; danielbernalconde@hotmail.com (L.D.B.-C.); veronicap.stem@gmail.com (V.P.-M.); ale.torres@stanford.edu (C.A.M.-T.); roy321lp@hotmail.com (R.R.-A.); franciscoj.padilla@iteso.mx (F.J.P.-G.); alexa_delgon@comunidad.unam.mx (A.D.-G.); margaritachavarin@gmail.com (M.G.-C.); 2 Molecular Neuropathology Department, Neuroscience Division, Institute of Cell Physiology, National Autonomous University of Mexico, Mexico City 04510, Mexico; 3 Department of Pathology, Stanford University School of Medicine, Stanford, CA 94304, USA; bschuele@stanford.edu; 4 Movement and Sleep Disorders Unit, Dr. Manuel Gea González General Hospital, Mexico City 14080, Mexico; ariasemc2@gmail.com; 5 Neurodevelopment and Physiology Department, Neuroscience Division, Institute of Cell Physiology, National Autonomous University of Mexico, Mexico City 04510, Mexico; marcelap@ifc.unam.mx; 6 National Laboratory of Genomic Resources, Institute of Biomedical Research, National Autonomous University of Mexico, Mexico City 04510, Mexico; collazo@iibiomedicas.unam.mx; 7 Laboratory of Molecular Pathogenesis, Laboratory 4, Building A4, Medical Surgeon Career, Faculty of Higher Studies Iztacala, National Autonomous University of Mexico, Mexico City 54090, Mexico; oskarsoto123@unam.mx

**Keywords:** alpha-synuclein, dopaminergic neurons, human induced pluripotent stem cells, cell transplant, 6-OHDA, Parkinson’s disease

## Abstract

Parkinson’s disease (PD) caused by *SNCA* gene triplication (3X*SNCA*) leads to early onset, rapid progression, and often dementia. Understanding the impact of 3X*SNCA* and its absence is crucial. This study investigates the differentiation of human induced pluripotent stem cell (hiPSC)-derived floor-plate progenitors into dopaminergic neurons. Three different genotypes were evaluated in this study: patient-derived hiPSCs with 3X*SNCA*, a gene-edited isogenic line with a frame-shift mutation on all *SNCA* alleles (*SNCA* 4KO), and a normal wild-type control. Our aim was to assess how the substantia nigra pars compacta (SNpc) microenvironment, damaged by 6-hydroxydopamine (6-OHDA), influences tyrosine hydroxylase-positive (Th+) neuron differentiation in these genetic variations. This study confirms successful in vitro differentiation into neuronal lineage in all cell lines. However, the *SNCA* 4KO line showed unusual LIM homeobox transcription factor 1 alpha (Lmx1a) extranuclear distribution. Crucially, both 3X*SNCA* and *SNCA* 4KO lines had reduced Th+ neuron expression, despite initial successful neuronal differentiation after two months post-transplantation. This indicates that while the SNpc environment supports early neuronal survival, *SNCA* gene alterations—either amplification or knock-out—negatively impact Th+ dopaminergic neuron maturation. These findings highlight *SNCA*’s critical role in PD and underscore the value of hiPSC models in studying neurodegenerative diseases.

## 1. Introduction

Parkinson’s disease (PD), the second most prevalent neurodegenerative disorder after Alzheimer’s disease [1], is clinically distinguished by the cardinal motor symptoms: tremor, rigidity, bradykinesia, and postural instability [2,3]. Additionally, non-motor symptoms (preceding by several years the movement symptoms in most patients) include sleep disorders, cognitive impairment (decline in memory, dementia and hallucinosis), autonomic dysfunction, and sensory symptoms (i.e., hyposmia) [2,3]. These manifestations contribute to progressive disability and an elevated risk of mortality [4,5,6]. The above is significant given that the prevalence of PD has increased worldwide [7]. Indeed, the analysis of Ou and colleagues estimated a global total of 8.5 million PD patients in 2019 [8].

PD has a multifactorial origin involving environmental (90–95% of cases) and genetic factors (5–10%), with at least eleven genes being the most common cause of PD (i.e., *GBA*, *PARKIN*, *DJ1*, *PINK1*, *LRRK2*, *CHCHD2*, *VPS35*, and *SNCA*) [7,9]. Determining the origin of PD is challenging given the long prodromal course of the disease [7]. Nowadays, imaging analysis such as magnetic resonance imaging, computed tomography scans, and positron emission tomography support the clinical diagnosis and prognosis of PD [10]. However, no biomarkers to diagnose the disease exist.

The distinctive pathological features of PD include the following: (i) dopaminergic neuron degeneration in the substantia nigra pars compacta (SNpc), leading to nigrostriatal pathway loss and reduced dopamine release to the putamen [11,12], and (ii) Lewy body formation, primarily comprising alpha-synuclein (α-syn), distributed in an ascending pattern as per Braak stages [13,14]. Under physiological conditions, α-syn is implicated in soluble N-ethylmaleimide-sensitive factor attachment protein receptor (SNARE)-complex assembly at presynaptic terminals [15]. However, misfolded and aggregated α-syn can detrimentally impact A9 midbrain dopaminergic neurons of the SNpc and gradually affect other areas of the brain, precipitating cell death and PD development [13,14,16,17]. The *SNCA* gene, encoding α-syn, was first linked to familial PD through point mutations [18]. In 2003, the genomic triplication of *SNCA* (3X*SNCA*) was identified and associated with an autosomal dominant, aggressive early-onset PD form [19].

Despite advancements in understanding α-syn pathology, models replicating synucleinopathy remain limited, particularly regarding dopaminergic cell differentiation. Human induced pluripotent stem cells (hiPSCs) have emerged as valuable tools in genetic PD modeling [20,21,22]. Indeed, hiPSCs with a two-fold overexpression of α-syn (genomic triplication on the mutant allele and one *SNCA* copy on the wild-type allele) given by the 3X*SNCA* genotype have been investigated for their potential to differentiate into the mesencephalic dopaminergic phenotype. However, conflicting evidence has been reported. For instance, while several studies indicate that the 3X*SNCA* genotype does not impair midbrain dopaminergic differentiation [23,24,25], Oliveira and colleagues reported that the overexpression of α-syn had an adverse effect on neuronal commitment and dopaminergic differentiation in cell culture [26]. This led to a decrease in the expression of tyrosine hydroxylase (Th), the rate-limiting enzyme of the dopaminergic pathway synthesis.

Conversely, complete *SNCA* deletion offers resistance to MPTP-induced neurotoxicity [27] and could potentially prevent synucleinopathy spread [28,29]. Chen and colleagues observed unaltered dopaminergic markers Th and FoxA2 co-expression in *SNCA*-deleted neurons from human embryonic stem cells (hESCs) [28]. Nonetheless, the effects of α-syn overexpression or deletion during early floor-plate differentiation remain less explored.

hESCs and hiPSCs are increasingly recognized for their potential in PD cell replacement therapies via transplantation into the striatum [30], and SNpc, in this last specific case to restore the nigrostriatal pathway in a more physiological manner [31,32,33,34,35,36]. Grafting stem cells at various maturation stages, including as floor-plate progenitors, has shown promise due to their high dopaminergic differentiation potential [36,37]. In this line, Brot and colleagues observed the generation of mature midbrain dopaminergic neurons with long-term survival and motor functionality recovery when floor-plate hiPSC progenitors were grafted into the murine SNpc [36]. Nonetheless, despite that dopaminergic neurons seem to arise efficiently, signs of slowly developing pathology have been observed in ventral mesencephalic (VM)-patterned dopaminergic progenitors (equivalent to floor-plate progenitors in cell culture) from 3X*SNCA* hiPSCs grafted into the striatum in a lesioned model of PD [25]. On the contrary, whether the grafted floor-plate hiPSC progenitors with a lack of the *SNCA* gene can differentiate into the dopaminergic lineage and resist the pathology in vivo when transplanted in the SNpc is unknown. In this regard, it has been reported that CRISPR/Cas9 *SNCA*-/- dopaminergic neurons derived from hESCs exhibit resistance to the formation of pS129-α-syn aggregates [28].

Moreover, S*NCA*-/- mice showed resistance to induced synucleinopathy [29]. Therefore, it is required to conduct further investigations focused on α-syn dosage using humanized disease models. This is crucial for gaining insights into the prodromal development of the disease, which could also contribute to the development of rational cell replacement therapies, particularly those based on *SNCA* knock-out autologous hiPSC transplants. Furthermore, whether 3X*SNCA* or *SNCA*-deleted hiPSCs can differentiate into a tyrosine hydroxylase-positive (Th+) lineage within the SNpc at the floor-plate stage requires further research [38,39,40].

In line with the above, our study seeks to evaluate the influence of α-syn overexpression and deletion on the differentiation of Th-expressing cells in hiPSC lines following transplantation into 6-OHDA-lesioned SNpc. We compared three hiPSC lines, a PD-patient-derived 3X*SNCA* line, an isogenic *SNCA* gene knock-out (KO) line (*SNCA* 4KO), and a wild-type line from an unaffected sibling [24,41]. Our findings highlight the SNpc’s supportive role in early Th differentiation but also reveal limitations imposed by *SNCA* alterations on maintaining the Th+ phenotype.

## 2. Results

### 2.1. In Vitro Differentiation of hiPSCs into Floor-Plate Progenitors

**Figure 1:**
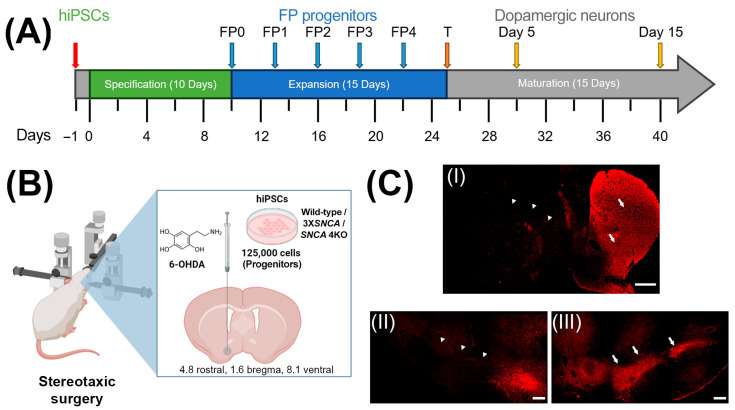
Overview of experimental methods. (**A**) In vitro dopaminergic differentiation protocol applied to wild-type, 3X*SNCA*, and SNCA 4KO hiPSC lines. (**B**) Stereotaxic 6-OHDA injection into left SNpc and transplantation of 125,000 hiPSCs at floor-plate stage (fifth expansion passage). (**C**) Representative coronal section images: (**I**) Striatal nucleus denervation in the left hemisphere (lesion site) shown by reduced tyrosine hydroxylase (Th, red signal) in the striatum (white triangles) contrasted with physiological Th expression in the contralateral striatum (white arrows) (right hemisphere). Scale: 1000 µm. (**II**) Similar denervation in the ipsilateral SNpc (white triangles) vs. (**III**) physiological Th expression in the uninjured contralateral SNpc (white arrows) (right side). Scale: 250 µm. Abbreviations: FP—floor-plate progenitor; T—transplant.

**Figure 2:**
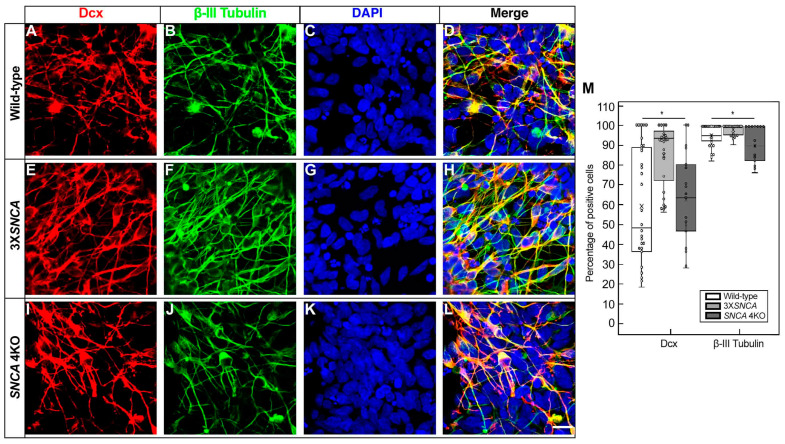
Doublecortin and β-III Tubulin expression in hiPSC-derived floor-plate progenitors. Representative images from wild-type (**A**–**D**), 3X*SNCA* (**E**–**H**), and *SNCA* 4KO (**I**–**L**) hiPSC lines at floor-plate progenitor stage corresponding to day 25 of differentiation. All lines show similar morphology and consistent expression of doublecortin (Dcx) (**A**,**E**,**I**) and β-III Tubulin (**B**,**F**,**J**), with comparable signaling patterns. Includes DAPI for nuclear staining. Scale bar: 15 µm. (**M**) Quantitative analysis of neuroblast (Dcx) and neuronal (β-III Tubulin) markers reveals a significant reduction in Dcx expression in the 3X*SNCA* line and a significant increase in β-III Tubulin expression in the *SNCA* 4KO line. Error bars indicate standard deviation. Abbreviations: Dcx (doublecortin), NS (not significant). * *p* < 0.01.

**Figure 3:**
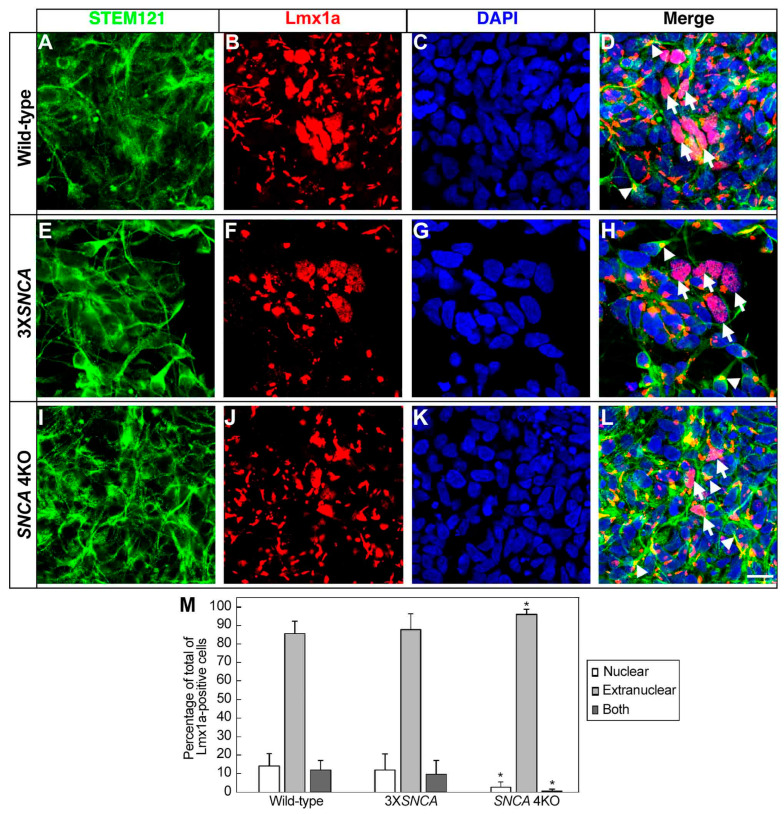
Lmx1a expression in hiPSC-derived floor-plate progenitors. Representative images from wild-type (**A**–**D**), 3X*SNCA* (**E**–**H**), and *SNCA* 4KO (**I**–**L**) hiPSC lines at floor-plate progenitor stage corresponding to day 25 of differentiation. Notably, wild-type (**B**) and 3X*SNCA* (**F**) lines demonstrate distinct Lmx1a signal patterns compared to *SNCA* 4KO (**J**). White arrowheads (**D**,**H**,**L**) indicate Lmx1a signals primarily near the axon hillock, contrasting with the dispersed nucleoplasm signals (white arrows, **D**,**H**,**L**). DAPI was used for nuclear staining. Scale bar: 15 µm. (**M**) Analysis of Lmx1a-positive cells shows a significant reduction in nuclear and extranuclear expression in the *SNCA* 4KO line compared to wild-type and 3X*SNCA* lines. Error bars indicate standard deviation. Abbreviations: Lmx1a (LIM homeobox transcription factor 1 alpha). * *p* < 0.01.

**Figure 4:**
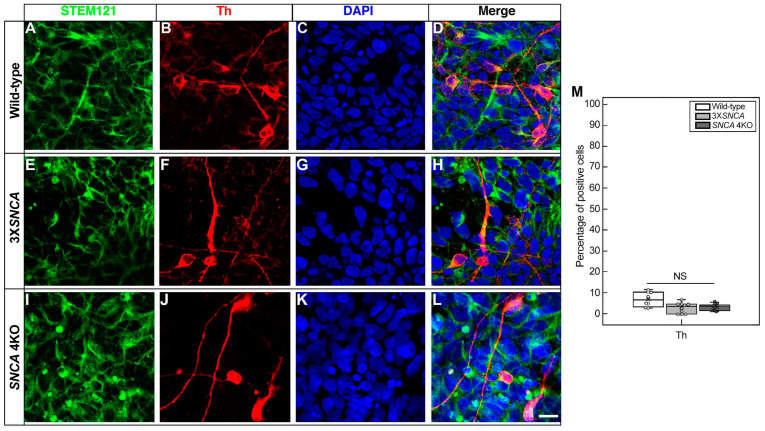
Tyrosine hydroxylase expression in hiPSC-derived floor-plate progenitors. Representative images from wild-type (**A**–**D**), 3X*SNCA* (**E**–**H**), and *SNCA* 4KO (**I**–**L**) hiPSC lines at floor-plate progenitor stage corresponding to day 25 of differentiation. Across these lines, a similar proportion of cells exhibit expression of the dopaminergic neuron marker tyrosine hydroxylase (Th) (**B**,**F**,**J**). DAPI was used for nuclear staining. Scale bar: 15 µm. (**M**) Quantitative analysis of dopaminergic (Th) marker reveals no significant differences in Th expression. Error bars indicate standard deviation. Abbreviations: Th (tyrosine hydroxylase), NS (not significant).

**Figure 5:**
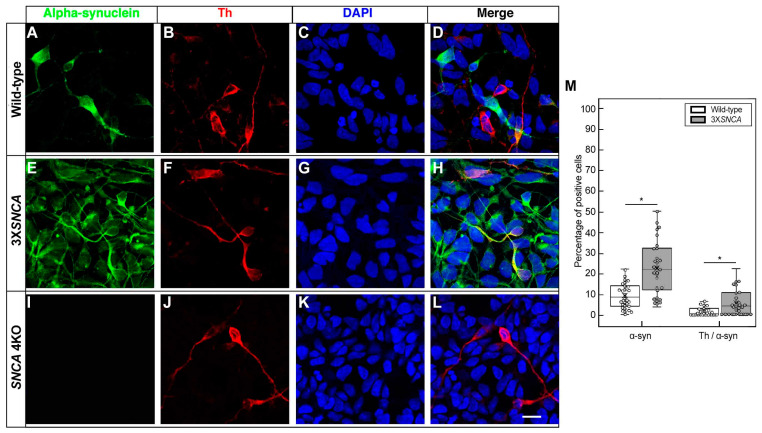
Tyrosine hydroxylase/alpha-synuclein expression in hiPSC-derived floor-plate progenitors. Representative images from wild-type (**A**–**D**) and 3X*SNCA* (**E**–**H**) hiPSC lines at floor-plate progenitor stage corresponding to day 25 of differentiation. A lower α-synuclein (α-syn) concentration is noted in the wild-type line (**A**–**D**) compared to the 3X*SNCA* line (**E**–**H**). The *SNCA* 4KO line (**I**–**L**), in contrast, shows no α-syn signal. DAPI was used for nuclear staining. Scale bar: 15 µm. (**M**) Quantification of α-syn and α-syn/Th co-expression highlights significant variations, especially in the 3X*SNCA* line. Error bars indicate standard deviation. Abbreviations: Th (tyrosine hydroxylase), α-syn (α-synuclein), NS (not significant). * *p* < 0.01.

First, we analyzed the impact of α-syn overexpression (3X*SNCA*) and complete deletion (*SNCA* 4KO) on the dopaminergic differentiation processes of hiPSCs, in comparison to wild-type hiPSCs. We used a protocol optimized for the specification and maturation of hiPSCs into dopaminergic neurons [36] (Figure 1) to examine the expression profiles of neuroblast, neuronal, and dopaminergic markers at the floor-plate stage (day 25 of differentiation) (Figure 1A, Figure 2, Figure 3, Figure 4 and Figure 5). Rats lesioned with 6-OHDA (Supplementary Table S1) were transplanted with the three hiPSC lines.

In vitro, positive co-expression for doublecortin (Dcx), a marker of neuronal progenitors [42] and immature neurons, and β-III Tubulin, indicative of the early and post-mitotic stage of neuronal differentiation [43], were demonstrated in the three cell lines at this stage (wild-type, Figure 2A–D; 3X*SNCA*, Figure 2E–H; and *SNCA* 4KO, Figure 2I–L). The statistical analysis (one-way ANOVA) revealed significant variations in Dcx expression among the cell lines (F(2,76) = [10.127], *p =* 0.0001) with the 3X*SNCA* line showing an increase compared to the wild-type and *SNCA* 4KO lines (Figure 2M). Similarly, significant differences were noted in β-III Tubulin expression (F(2,76) = [12.311], *p <* 0.0001), with the *SNCA* 4KO line displaying a decrease, as determined by Tukey’s HSD tests (Figure 2M). These results suggest distinct neuronal differentiation pathways among the cell lines. 

Subsequently, the expression of LIM homeobox transcription factor 1 alpha (Lmx1a), essential for the development of mesencephalic dopaminergic neurons, was evaluated (Figure 3) [44]. Using double immunofluorescence with STEM121, a human cytoplasmic protein-specific antibody, we noted no significant differences in the total Lmx1a-positive cells among the wild-type (Figure 3A–D), 3X*SNCA* (Figure 3E–H), and *SNCA* 4KO (Figure 3I–L) progenitors (F(2,80) = [4.3436], *p =* 0.0162) (Figure 3M). However, the *SNCA* 4KO line displayed a unique Lmx1a signal distribution, predominantly near the axon hillock and outside the nucleus (compare Figure 3D,H with Figure 3L, white arrows) (F(2,19) = [11.507], *p =* 0.0005; F(2,19) = [9.6304], *p =* 0.0013) (Figure 3M).

Despite these variances, the expression of Th, the rate-limiting enzyme in the synthesis of catecholamines and last stage in the dopaminergic differentiation [45], showed a comparable amount of positive cells across all cell lines: wild-type (Figure 4A–D), 3X*SNCA* (Figure 4E–H), and *SNCA* 4KO (Figure 4I–L) (F(1,16) = [0.1247], *p =* 0.7286) (Figure 4M). Conversely, the distinguishing feature of PD, α-syn expression, as expected, was significantly lower (F(1,58) = [23.0571], *p <* 0.0001) in the wild-type line compared to 3X*SNCA*, with no signal detected in *SNCA* 4KO (Figure 5). Moreover, the colocalization between Th and α-syn was significantly higher in the 3X*SNCA* line (F(1,58) = [11.2159], *p =* 0.0014), with numerous cells showing either α-syn positivity/Th negativity or vice versa (Figure 5D,H,M).

Additionally, we assessed the persistence of dopaminergic (Lmx1a+, Th+) and neuronal (Dcx+/β-III Tubulin+) expression patterns across the wild-type, 3X*SNCA*, and *SNCA* 4KO lines during days 5 and 15 of the dopaminergic maturation phase (day 30 and 40, respectively, of the complete cell culture) (Supplementary Figures S1 and S2). On day 15 of the maturation stage, the number of Th+ cells was similar among the wild-type (54.03 ± 4.61%), 3X*SNCA* (46.66 ± 10.19%), and *SNCA* 4KO (44.07 ± 10.47%) lines and had a robust increase compared to the floor-plate stage (Figure 4) due to the terminal maturation. Remarkably, the non-nuclear Lmx1a signal in the *SNCA* 4KO line continued at both maturation stages (Supplementary Figures S1C,F,I and S2C,F,I), as shown on day 15 of maturation: wild-type (73.43 ± 23.76%), 3X*SNCA* (70.84 ± 18.24%), and *SNCA* 4KO (74.10 ± 12.44%). 

### 2.2. Survival and Neuronal Maturation of hiPSCs in the SNpc

**Figure 6:**
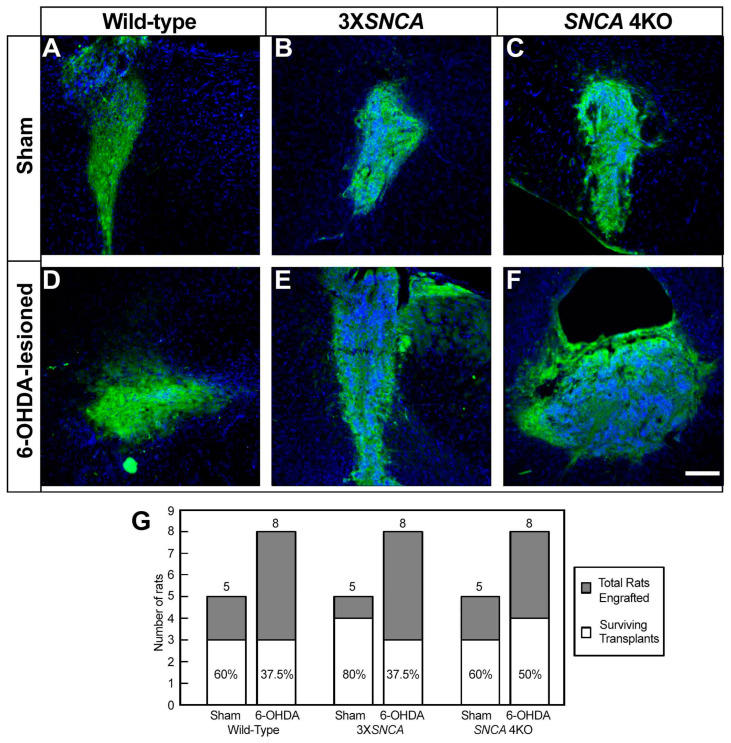
Survival of transplanted hiPSC-derived floor-plate progenitors at two months post-transplantation. Representative photomicrographs of wild-type (**A**,**D**), 3X*SNCA* (**B**,**E**), and *SNCA* 4KO (**C**,**F**) hiPSC lines under sham (**A**–**C**) and 6-OHDA-lesioned conditions (**D**–**F**). The images illustrate the survival of these cell lines in both SNpc conditions, assessed using the human cell marker STEM121 (green). DAPI (blue) was used for nuclear staining. Scale bar: 250 µm. (**G**) Percentage of rats with floor-plate surviving transplants at two months post-transplantation (mpt) and the number of rats grafted. The data show the % of rats out of the total transplanted (*n* = 39), showing graft survival via a positive STEM121 signal both in sham and 6-OHDA-lesioned SNpc. Cell survival was evaluated in at least three brain slides for each rat. Survival % (percentage of rats with graft survival), *n* (total number of transplanted rats).

Our study was extended to assess the survival and maturation capacity of floor-plate hiPSC progenitors with altered *SNCA* expression upon implantation in the SNpc. STEM121 immunofluorescence confirmed the robust survival of transplants from the wild-type, 3X*SNCA*, and *SNCA* 4KO hiPSCs at two months post-transplantation (mpt), regardless of the SNpc, irrespective of the expression level of the *SNCA* gene (Figure 6). The STEM121-positive signal also confirmed graft survival in both non-lesioned (Figure 6A–C) and lesioned (Figure 6D–F) SNpc environments. However, a higher percentage of rats with surviving transplants was observed in the sham (non-lesioned) condition compared to the lesioned SNpc (Figure 6G).

**Figure 7:**
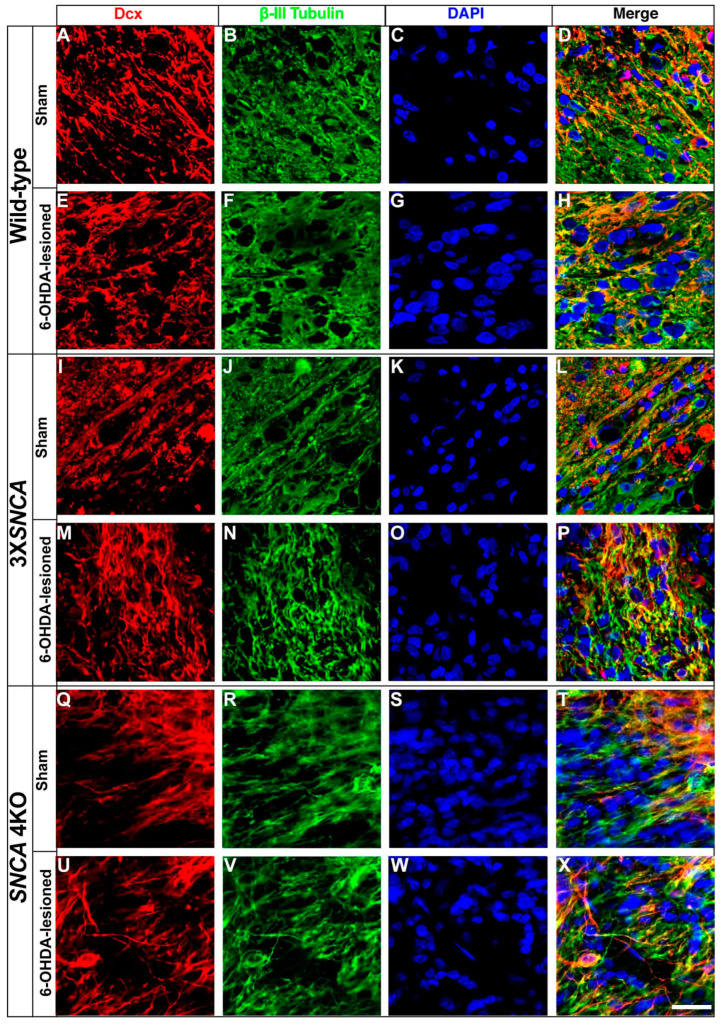
Post-transplant doublecortin and β-III tubulin expression in hiPSC-derived floor-plate progenitors**.** Representative images from wild-type (**A**–**H**), 3X*SNCA* (**I**–**P**), and *SNCA* 4KO (**Q**–**X**) hiPSC lines two months post-transplantation. All three lines exhibit positive staining for doublecortin (Dcx) (**A**,**E**,**I**,**M**,**Q**,**U**) and β-III Tubulin (**B**,**F**,**J**,**N**,**R**,**V**) in both sham and 6-OHDA models, suggesting ongoing neuronal maturation. DAPI was used for nuclear staining. Scale bar: 15 µm.

To evaluate neuronal maturation, we performed double immunolabeling for Dcx and β-III Tubulin in tissue slices adjacent to those analyzed for STEM121. At two mpt, all three hiPSC lines (wild-type, 3X*SNCA*, and *SNCA* 4KO) showed robust labelling for both Dcx and β-III Tubulin markers in both sham and 6-OHDA-lesioned rats (Figure 7). This suggests that the SNpc is permissive to maintaining the neuronal phenotype of the hiPSC lines at the floor-plate stage. 

### 2.3. Th+ Differentiation in the Lesioned SNpc

**Figure 8:**
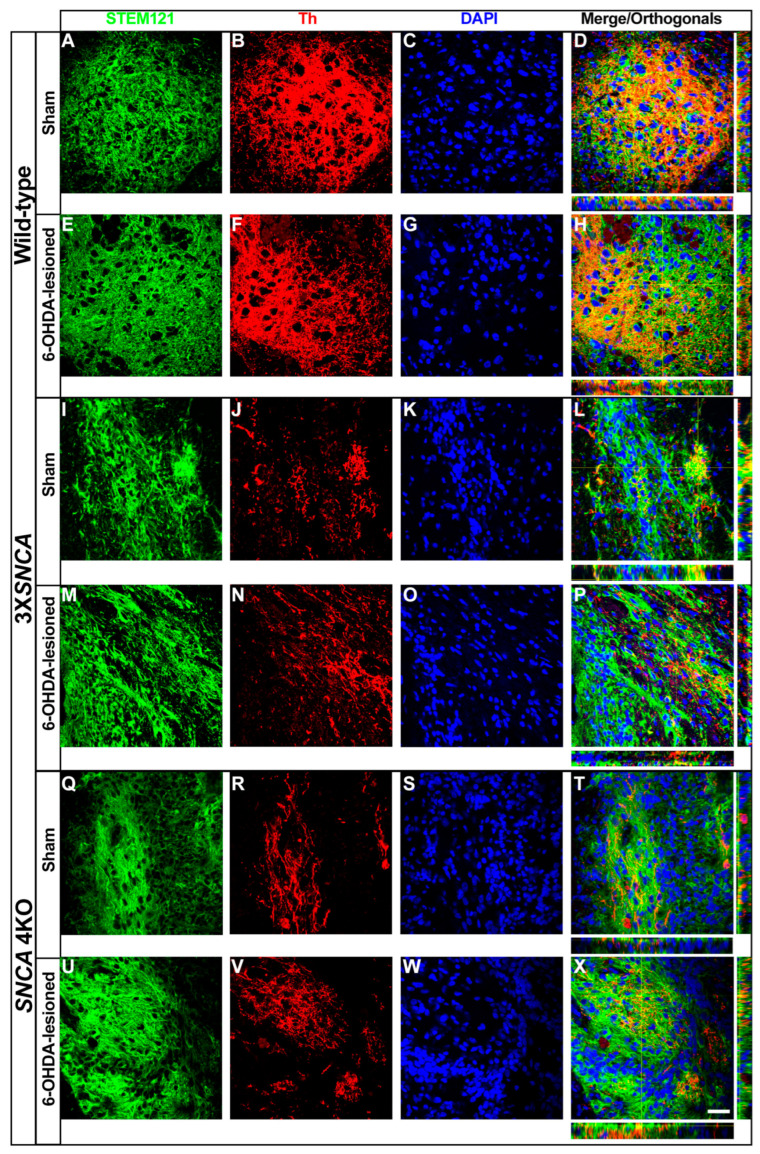
Tyrosine hydroxylase expression in transplanted hiPSC-derived floor-plate progenitors at two months post-transplantation. Representative images of wild-type (**A**–**H**), 3X*SNCA* (**I**–**P**), and *SNCA* 4KO (**Q**–**X**) hiPSC lines. These images show Th expression in cells transplanted at the floor-plate phase, assessed two months post-transplantation, in the small regions where the Th signal was observed. The human cell marker STEM121 was utilized to identify human transplanted cells. Notably, a reduction in Th+ signal was observed in both 3X*SNCA* (**J**,**N**) and *SNCA* 4KO (**R**,**V**) lines. In orthogonal views (**D**,**H**,**L**,**P**,**T**,**X**), cross-sections emphasize regions of intense factor colocalization. Scale bar: 30 µm. Abbreviations: Th (tyrosine hydroxylase).

We previously demonstrated that the 6-OHDA-lesioned SNpc favors the Th+ fate of grafted embryoid body cells (EBCs) derived from mouse ESCs and human EBCs overexpressing dopaminergic transcription factors [39,40]. To assess the Th+ lineage maturation potential of 3X*SNCA* and *SNCA* 4KO floor-plate progenitors in the SNpc, we examined the Th expression compared to the wild-type line. While the Th-intermingled pattern displayed by the grafted precludes the quantification of Th+ cells, it was still feasible to observe the colocalization of Th+ cells with STEM121 in the wild-type at two mpt (Figure 8A–H) and in discrete regions of the 3X*SNCA* and *SNCA* 4KO transplanted hiPSCs (Figure 8I–X and Supplementary Figure S3 in a minor magnification). However, both 3X*SNCA* and *SNCA* 4KO lines exhibited an evident decrease in the Th+ expression (Figure 8I–X). Notably, the Th+ signal in the sham condition appeared similar to that in the 6-OHDA-lesioned SNpc, indicating that the lesion did not enhance Th+ differentiation in these hiPSC progenitors. 

### 2.4. Alpha-Synuclein Expression in Grafted hiPSCs

**Figure 9:**
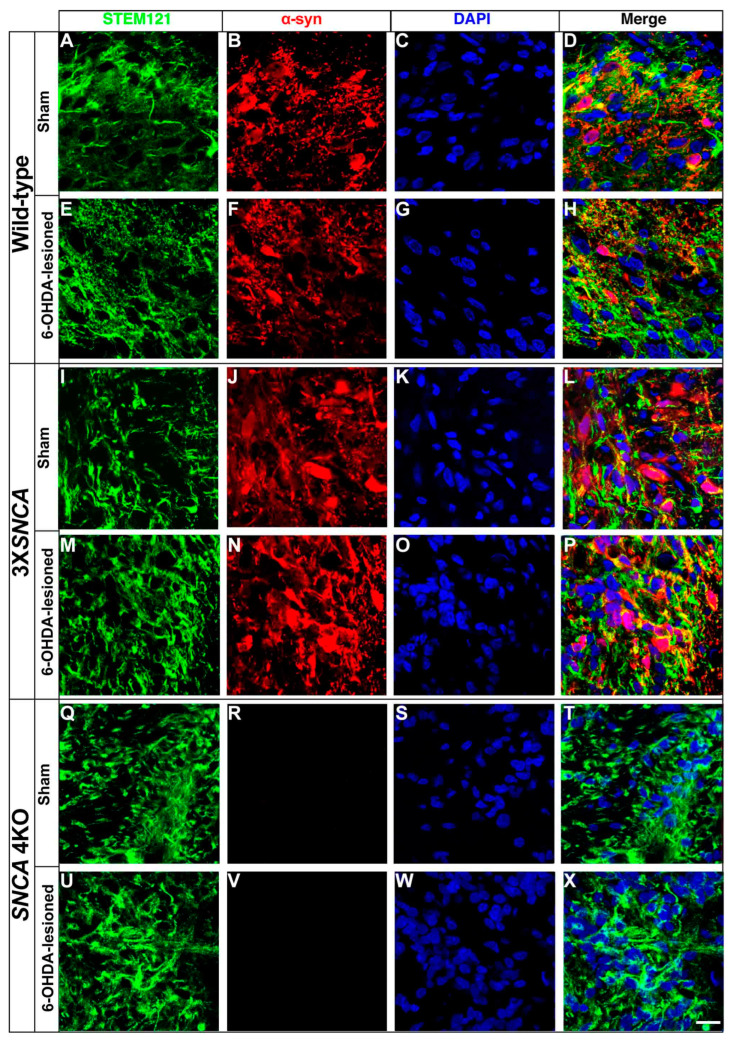
Alpha-synuclein expression in transplanted hiPSC-derived floor-plate progenitors at two months post-transplantation. Representative images from wild-type (**A**–**H**), 3X*SNCA* (**I**–**P**), and *SNCA* 4KO (**Q**–**X**) hiPSC lines. These images depict α-syn expression in cells transplanted at the floor-plate phase, assessed two months post-transplantation. Notably, an increase in α-syn is seen in the 3X*SNCA* line (**J**,**L**,**N**,**P**), compared to the wild-type (**B**,**D**,**F**,**H**), while no expression is observed in the *SNCA* 4KO line (**R**,**T**,**V**,**X**). Scale bar: 15 µm. Abbreviations: α-syn (alpha-synuclein).

Next, we evaluated whether α-syn expression, a neuropathological hallmark of PD, influences the development of the Th+ phenotype in the transplanted hiPSCs in the SNpc. At two mpt, α-syn expression colocalized with STEM121 in sham and injured wild-type rats (Figure 9A–H). The 3X*SNCA* line showed an increased α-syn signal in both sham and lesioned conditions, with a notable intensification in the lesioned SNpc (Figure 9I–P). In contrast, the *SNCA* 4KO line showed no α-syn signal (Figure 9Q–X), highlighting the absence of α-syn in these cells. These results indicate that α-syn overexpression in the 3X*SNCA* line is accentuated in the lesioned SNpc environment, suggesting a potential interaction with the injury-induced microenvironmental changes.

## 3. Discussion

This work investigated the critical role of α-syn dosage in developing and maintaining the Th/Lmx1a expression markers, essential for the dopaminergic phenotype, which is relevant to understanding PD neurodegeneration. We examined a human induced pluripotent stem cell (hiPSC) line from a PD patient with the 3X*SNCA* mutation and its isogenic *SNCA* 4KO line, focusing on the capacity of floor-plate progenitor cells to develop the Th+ lineage. Our cell culture protocol was effective since we obtained many Lmx1a floor-plate progenitors and Lmx1a+/ Th+ on mature neurons from hiPSCs. The differentiation procedure for inducing the Th+ phenotype is similar to the protocol described by Kirkeby and colleagues, wherein many ventral midbrain progenitors are derived from hESCs [36,46]. Here, the 3X*SNCA* line showed a significant increase in α-syn expression compared to the wild-type line (Figure 5E,H), consistent with previous research [26,41]. Conversely, as expected, the SNCA 4KO line did not exhibit α-syn expression (Figure 5I,L), which confirms our earlier findings [41,47] and those of others [26].

Our findings revealed that all three cell lines developed a robust Dcx+/β-III Tubulin+ phenotype at the floor-plate stage (Figure 2), indicating their status as neuroblasts and immature neurons [48]. However, the 3X*SNCA* line showed a notable increase in Dcx+ cells yet a decrease in β-III Tubulin expression (Figure 2E–H), compared to the wild-type and *SNCA* 4KO lines (Figure 2A–D,I–L). This suggests that α-syn overexpression may differentially affect markers of neural induction and neuronal differentiation. 

In this study, as anticipated, the cells did not fully adopt a mature Th+ phenotype at the floor-plate stage. This was supported by the lower percentage of Th+ cells observed across all lines (Figure 4), a marker known to increase as dopaminergic differentiation progresses [36], as occurred in our cell culture protocol, where the number of Th+ cells increased after 40 days of the differentiation period (Figure S2G–I). Nevertheless, a notable abundance of Lmx1a+ cells was observed (Figure 3), highlighting the presence of this crucial transcription factor essential for dopaminergic specification, as it is a positive regulator of NURR1 and Pitx3 expression, which in turn are positive regulators of Th expression [44,49,50,51,52]. Interestingly, unlike the wild-type line, the Lmx1a expression pattern was predominantly non-nuclear in the *SNCA* 4KO line (Figure 3M). This suggests an alteration in this particular dopaminergic marker only during the specification period since the recognized nuclear pattern was recovered at the maturation stage. Our results in the maturation stage are in agreement with others; notably, the deletion of the endogenous *SNCA* gene did not affect the efficiency of hESC differentiation into dopaminergic neurons [28], although in that study, other dopaminergic markers that did not include Lmx1a were analyzed by RT-PCR. In this respect, no significant differences in neuronal development have been reported in α-syn null mouse models, although specific markers of dopaminergic development were not analyzed [53,54].

The observed cellular localization of Lmx1a indicates a potential “relationship” with the Golgi complex, especially considering that the antibody used recognizes Lmx1 in this organelle. Thus, additional research is necessary to confirm this potential association. This finding is particularly relevant considering that a previous study reported a significant reduction in the number of dopaminergic neurons within the SNpc during development (E13.5) in α-syn null mouse models [55], which suggests that alpha-synuclein is required for the survival or maturation of dopaminergic neurons in the developing SNpc. Therefore, assessing dopaminergic markers involved in the specification of the dopaminergic lineage, as in the present study, may give a more profound insight into the impact of α-syn in the dopaminergic lineage development. We are particularly interested in following the effects of α-syn in the specification of this phenotype.

On the other hand, we observed that the non-nuclear Lmx1a pattern in the *SNCA* 4KO line was altered on day 15 of maturation in vitro (Figure S2G–I), where it resembled the nuclear pattern seen in the wild-type line. This suggests that the absence of α-syn plays an essential role by influencing the expression pattern of Lmx1a during the early differentiation phase. However, the Lmx1a+ and Th+ cells’ total count remains consistent across all differentiation stages, regardless of *SNCA* dosage. The implications of this expression pattern shift during Th+ neuronal development have not been elucidated, and research is required. 

Previous studies have linked α-syn overexpression associated with genetic PD to adverse effects on neuronal commitment and Th differentiation in cell culture [26]. However, other research suggests that the 3X*SNCA* genotype does not impede the differentiation of midbrain dopaminergic cells [23,24,25]. Our results support the latter, indicating that the triplication of the *SNCA* gene locus encoding α-syn does not modify the total number of Th+ cells in vitro. However, it does affect Lmx1a subcellular localization. Moreover, it confers susceptibility to stressors like reactive oxygen species, glucose deprivation, and neurotoxins, potentially leading to cell degeneration and death [16,26,56,57]. This aligns with findings that α-syn toxicity can be reversed in mutation-corrected neurons derived from Parkinson patient hiPSCs or with reduced *SNCA* mRNA levels [58,59]. Additionally, dysregulated dopamine release and firing activity in 3X*SNCA* cells, restored with D2 receptor agonist quinpirole administration, further highlight the complex role of α-syn in PD pathology [60]. However, the effects of the 3X*SNCA* or *SNCA* gene absence in in vivo models remain less explored, underlining the need for additional investigation.

The primary objective of this study was to assess the potential of these hiPSCs to differentiate efficiently into Th+ cells in the SNpc despite variations in α-syn expression. In line with emerging cell replacement therapies for PD [61,62], our findings are crucial for understanding the feasibility and efficacy of such approaches. Despite the successful optimization of cell transplantation for dopamine release in animal models, particularly in the striatum, the challenge remains in achieving functional restoration of the host nigrostriatal pathway when cells are transplanted into the SNpc [22]. In this regard, the engraftment of progenitor cells derived from hESCs or hiPSCs has demonstrated the feasibility of functional integration [37]. Additionally, others have reported the functional integration of progenitors or midbrain dopaminergic neurons derived from hESCs and hiPSCs implanted in the SNpc [31,32,33,34,35,36]. These results strongly suggest the feasibility of the survival and effective differentiation of dopaminergic neurons derived from dopaminergic progenitor cells in the SNpc. Our data demonstrate that the intact rat SNpc is a permissive neurogenic environment for neuronal development [38,39,40]. Yet, it does not inherently promote the mesencephalic dopaminergic phenotype without the overexpression of key dopaminergic transcription factors like Lmx1a [39,40]. This emphasizes the importance of understanding the microenvironmental influences on transplanted cells.

After identifying the neural, neuronal, and dopaminergic marker expression of the wild-type, 3X*SNCA*, and *SNCA* 4KO cell lines, our transplantation experiments in a preclinical model of 6-OHDA revealed robust cell survival across different cell lines at the floor-plate stage (Figure 6), with no excessive cell division or even tumor formation in the transplants [63,64,65]. Notably, the grafted 3X*SNCA* line exhibited a marked increase in the α-syn signal (Figure 9J,N), while the *SNCA* 4KO line showed an absence (Figure 9R,V). Despite maintaining a comparable immature neuron state (β-III Tubulin+/Dcx+) in both sham and lesioned SNpc of rats (Figure 7), a significant reduction in Th expression was observed in the 3X*SNCA* (Figure 8J,N) and *SNCA* 4KO (Figure 8R,V) lines’ intranigral grafts. This suggests that the overexpression or absence of α-syn limits the potential of Th differentiation in Lmx1a+ lineage-committed floor-plate progenitor cells. In this regard, a significant dopamine decrease was observed in the striatum of rats overexpressing α-syn [66] or mutant mice lacking α-syn [67,68], concomitantly with the reduction in Th+ innervation in the striatum [66,67]. Our transplants are similar to the previous report where efficient Th+ differentiation into the SNpc was observed using hiPSCs, although in our case, not robust Th+ fiber outgrowth was observed [36]. Also, it shows a difference in the efficient maturation of VM-patterned DA progenitors from a 3X*SNCA* line (AST18) into Th+ cells transplanted in the striatum of 6-OHDA-lesioned rats [25]. However, we cannot dismiss that these differences could be due to the genetic variations among patient-derived cells that could affect the cellular phenotype [69] and, consequently, the differentiation of the transplanted cells. 

It is to be noted that, as a preclinical study, the results of our analysis exhibit limitations associated with the intrinsic nature of the research. For instance, the observed survival rates under our transplantation conditions could potentially be augmented through the implementation of alternative cyclosporin A treatment regimens or the utilization of immunodeficient mouse or rat models: such approaches could mitigate the limitations associated with graft survival, as previously reported [70], thereby facilitating further investigation into differentiation within the SNpc over an extended period. Similarly, additional research is required to confirm terminal neuronal differentiation, including assessing differentiation markers such as microtubule-associated protein 2 (MAP2) [71]. Additionally, longer-term studies are necessary to evaluate the impact of the transplantation, particularly on the functional integration of the nigrostriatal circuit evaluated by motor recovery and dopamine release [33]. A recent groundbreaking study by Schweitzer and colleagues has achieved a significant milestone by transplanting autologous hiPSC-derived dopaminergic progenitors into the striatum of a PD patient, showing promise as a potential therapeutic strategy [61]. Utilizing the patient’s cells ensures immunological compatibility and eliminates the need for immunosuppression. In this regard, utilizing cells bearing a deletion of *SNCA* has emerged as a potential transplantation option, as neurons lacking α-syn, engineered from stem cells, demonstrate resistance to synucleinopathy [27,28,29]. Therefore, our findings offer valuable insights for developing rational and successful personalized stem cell-based therapies targeting α-syn dosage. In line with the above, our cell-based study allows us to understand the progression of the disease in the early stages, which might also facilitate further research on disease-modifying therapies capable of avoiding the progression of PD.

Our study comprehensively analyzes the role of α-syn in Th+ neuronal differentiation using hiPSC models derived from PD patients. The findings highlight the complexity of α-syn’s impact on PD pathology and offer valuable insights for developing effective stem cell-based therapies. While the transplantation of hiPSC-derived dopaminergic progenitors shows promise, our results suggest that variations in α-syn expression can influence the differentiation of Th+ cells in vivo. However, the variability in genetic variations among patient-derived cells can also be considered. Future research should continue to explore the nuanced interactions between α-syn, dopaminergic neuron differentiation, and the microenvironmental factors within the SNpc to optimize cell-based therapies for PD. Furthermore, our study results prompt further investigation into the role of α-synuclein in both overexpression and underexpression scenarios, particularly concerning its impact on dopaminergic lineage specification.

Additionally, we aim to explore how α-synuclein alterations affect differentiation in oligodendroglial and neuronal lineages in synucleinopathies like Multiple System Atrophy and Lewy body disease, areas that have received less attention. Although hiPSCs exclusively from α-synuclein-altered patients are currently unavailable, ongoing initiatives suggest promising avenues for future research. This research area remains open, offering significant potential to advance our understanding of α-synuclein’s role in neurodegenerative diseases.

## 4. Materials and Methods

### 4.1. Human Induced Pluripotent Stem Cell Lines and CRISPR-Engineered hiPSCs

Three cell lines developed and exhaustively characterized by Zafar and colleagues [41] and Byers and colleagues [24] were used in this study. All methods were carried out in accordance with relevant guidelines and regulations. All experimental protocols were approved by the Institutional Animal Care and Use Committee of the Institute of Cell Physiology (permit number: MGC65-19) of the National Autonomous University of Mexico. Written informed consent was obtained from all subjects and/or their legal guardian(s) involved in the Zafar and colleagues [41] and Byers and colleagues [24] studies where the hiPSC lines were generated and first described. The cell lines were as follows: (i) hiPSCs derived from a PD patient with the 3X*SNCA* mutation (PI-1754, four alleles), (ii) its isogenic genetically engineered *SNCA* 4KO (*SNCA*3X_4KO_C1, 0 alleles), and (iii) as a control, a wild-type hiPSC line without the 3X*SNCA* mutation from an unaffected sibling of the 3X*SNCA* patient (PI-1761, two alleles). In brief, primary adult human dermal fibroblasts were obtained from a male with PD due to the 3X*SNCA* mutation and his unaffected sister, who possessed an average copy number of the *SNCA* gene. The cell lines were then reprogrammed via induced expression of the OCT4, SOX2, KLF4, and c-MYC transcription factors. The resultant clones displayed positivity for pluripotency-associated antigens, including SSEA3, TRA1-60, TRA-181, NANOG, and SSEA4, while exhibiting negativity for the SSEA1 marker [24]. The knock-out cell line was generated by Zafar and colleagues [41]. The CRISPR/Cas9 gene editing system was employed, targeting exon 2 of each of the four alleles of the *SNCA* gene from the PD patient with the *SNCA* gene triplication. This targeted approach led to the elimination of protein expression across all four alleles.

### 4.2. Cell Culture

In vitro, differentiation was performed following the three-step protocol of the PSC Dopaminergic Neuron Differentiation Kit (Thermo Fisher Scientific, Gibco, Waltham, MA, USA, A3147701) with some modifications. This protocol has previously been documented for its capacity to yield highly efficient and reproducible floor-plate dopaminergic progenitors and facilitate dopaminergic differentiation [36]. Firstly, hiPSCs were thawed in 0.1% gelatin-coated 6-well culture plates (Sigma Aldrich, St. Louis, MO, USA, and Thermo Fisher Scientific, respectively) with mouse embryonic fibroblasts as feeder cells in hiPSC medium (DMEM-F12, KnockOut Serum Replacement, GlutaMAX, nonessential amino acids, penicillin–streptomycin, β-mercaptoethanol, and Fgf2) [72]. Once colonies were present, they were transitioned to a feeder-free culture. Cell clusters were manually harvested and seeded on feeder-free geltrex 1%-coated plates (Thermo Fisher Scientific) with 2 mL of STEMFLEX medium (Gibco). After cell monolayer formation, manual passage was performed every 6–7 days for cell banking or enzymatic dissociation with Accutase (Thermo Fisher Scientific), considering 1 mL per 10 cm^2^ of surface area (hereafter, in each passage, Accutase was used). 

Next, a 10-day specification phase was started using Vitronectin (1 μg/cm^2^, Gibco)-coated plates with 2 mL specification medium (Gibco, A31468-01) to generate floor-plate progenitors. The specification medium was replaced every two days. After, a 15-day expansion step was performed using laminin (1 μg/cm^2^, Gibco)-coated plates with 2 mL expansion medium (Gibco, A3165801) to increase the number of floor-plate progenitors. During the expansion phase, cell passaging occurred every three days over five cycles. Cryopreservation of floor-plate progenitors started from the second passage onwards. In each subculture, a 5 mm ROCK inhibitor Y27632 (Thiazovivin, Stemgent, MA, USA) was exclusively added for the initial 24 h. Following this, for the next 48 h, an expansion medium was added without the ROCK inhibitor. 

Upon reaching the fifth passage, the cells were transplanted at the floor-plate phase (day 25 of differentiation) or continued with their in vitro maturation stage. The last culture phase consisted of the use of poly-D-lysine (10 μg/cm^2^, Sigma)/laminin (1.5 μg/cm^2^)/Geltrex triple-coated plates with 2 mL maturation medium DMEM-F12 (Gibco), with maturation supplement (Gibco, A31474-01) for 5 or 15 days for in vitro analysis. Thiazovivin was supplemented throughout the protocol at each thawing or passing, 1.5 µM and 1 µM, respectively. 

### 4.3. Animals

Adult male Wistar rats (8–9 weeks of age) weighing 250 g were used in this study. They were housed under standard vivarium conditions: 12:12 light/dark cycle, controlled room temperature (27 °C), and *ad libitum* access to food and water. All methods were carried out in accordance with relevant guidelines and regulations (National Institute of Health Guide for the Use and Care of Laboratory Animals). All experimental protocols were approved by the Institutional Animal Care and Use Committee of the Institute of Cell Physiology (permit number: MGC65-19) of the National Autonomous University of Mexico. Animal numbers were kept to a minimum, and all efforts were made to reduce animal suffering. All techniques are described according to ARRIVE’s guidelines for reporting animal research [73]. Groups were evaluated at two mpt.

### 4.4. Rodent Model of Parkinson’s Disease

A PD model was generated through unilateral administration of the neurotoxin 6-hydroxydopamine (6-OHDA), which effectively targets and kills catecholaminergic neurons [74]. Following our previous work [39,40], rats were given intraperitoneal anesthesia using a mixture of xylazine (8 mg/kg, PiSA, Mexico City, Mexico) and ketamine (100 mg/kg, PiSA) before surgical procedures. Of the total of 39 anaesthetized animals, 24 (6-OHDA group) and 15 (Sham group) were secured in a stereotaxic device and injected with a total volume of 0.5 mL of a 0.9% saline solution supplemented with 40 μg of 6-OHDA (Sigma-Aldrich) and 32 μg/mL of L-ascorbate (JT Baker, Phillipsburg, NJ, USA) into the left SNpc at a rate of 0.125 mL/min [39,40]. The coordinates for the injection site were −4.7 mm AP, ±1.6 mm ML, and −8.2 mm DV relative to Bregma 0 [75,76]. The 6-OHDA group was compared to a control group of 15 sham rats that underwent the same procedure but received a 0.9% saline solution instead of 6-OHDA.

### 4.5. Rotational Behavior

Two weeks after injury, animals were injected with amphetamine intraperitoneally (4 mg/kg, Sigma Aldrich) to measure dopaminergic imbalance and confirm dopaminergic depletion in the nigrostriatal pathway, as previously described [40,75]. Using a customized computerized image and movement recognition system already reported by our group [40,75], the number of left and right turns was counted for 90 min. Only animals having >400 rotations ipsilateral to the 6-OHDA lesion were included in the tests. Animals were assessed two weeks following the lesion.

### 4.6. Transplant Procedure

As we previously reported, 15 days after 6-OHDA infusion, the lesion stabilized [39,40]: at this moment, the animals were anaesthetized using the method described earlier for cell transplantation. The three cell lines were grafted at the floor-plate expansion stage (in FP4 at day 25). The exact stereotaxic coordinates and anesthetic concentrations mentioned above were used in the injured or sham rats for transplantation of approximately 125,000 floor-plate cells resuspended in 3 µL of PBS (preparation described in the culture section) using a 5 μL Hamilton syringe (Thomas Scientific, Swedesboro, NJ, USA). The cannula remained in place for at least five minutes after the 6-OHDA or cell injection was finished to prevent backflow. To prevent graft rejection, transplanted rats were subjected to daily oral cyclosporine A (10 mg/kg, PiSA) treatment until brain removal, starting one day before transplantation.

### 4.7. Euthanasia of the Animals and Fixation of Brains

At two mpt, rats were deeply anaesthetized with an intraperitoneal injection of 1 mL 6.3% sodium pentobarbital (60 mg/kg, PiSA) and perfused intracardially with 200 mL of 0.1 M phosphate buffer (Baker) followed by 100 mL 4% paraformaldehyde (PFA, Millipore, MA, USA). Brains were postfixed overnight in 4% PFA and cryoprotected by sequential 24 h washes with 10%, 20%, and 30% sucrose in phosphate-buffered saline (PBS, Thermo Fisher Scientific). The brains were then sliced with a cryostat at 40 µm in the coronal plane and collected in PBS. Finally, the slices were cryopreserved at 4 °C using an antifreeze solution (glycerol/ethylene glycol/PBS).

### 4.8. In Vitro Immunofluorescence

Immunofluorescence of cell cultures in 8-well chamber slides (Nunc Lab-Tek, Thermo Fisher Scientific) was performed according to the Human Dopaminergic Neuron Immunocytochemistry Kit (Invitrogen, A29515). Cells were fixed and incubated following the protocol of the kit; subsequently, the primary antibody (Supplementary Table S3) diluted in blocking solution was added to the well chamber for 3 h at room temperature, followed by the addition of the secondary antibody (Supplementary Table S3) diluted in blocking solution for 1 h at room temperature. After secondary antibody incubation, for staining cell nuclei, the fluorescent DNA dye 4′,6-diamidino-2-phenylindole (DAPI, 1:10,000; Biostatus Limited, Loughborough, UK) was added, incubated for 5 min, and rinsed three times in PBS. Finally, ProLong Diamond Antifade Mountant (Invitrogen) was applied for prolonged storage.

### 4.9. Ex Vivo Immunofluorescence

As we previously indicated [40], for immunofluorescence, brain slices were washed three times in PBS for 10 min, then rinsed three times for 10 min in 0.3% Triton X-100 PBS, treated with antigen retrieval citrate buffer (10 mM, pH 6.1, BioSB, Santa Barbara, CA, USA) at 65 °C for 35 min, washed three times with PBS, and incubated with primary antibodies (Supplementary Table S3) diluted in blocking solution (10% bovine serum albumin in PBS) overnight at 4 °C. After washing in PBS, the slices were incubated for two hours at room temperature with the corresponding secondary antibody (Supplementary Table S3). Finally, the sections were rinsed three times with PBS, and DAPI DNA dye (prepared as outlined in the preceding section) was added to the final rinse for staining of cell nuclei. Each brain slice was mounted following the procedure detailed previously. 

### 4.10. Microscopy and Cell Counting

Images of cultures and tissues were acquired using a Zeiss LSM800 inverted confocal microscope, Olympus FV1000 multi-photon confocal microscope (Upright BX61WI and inverted IX81), and Leica DM6000B epifluorescence microscope. ImageJ/FIJI 1.54 software was used for image processing, and the CookBook extension was used to count the different markers in cell count. In total, 3–5 fields of 3 independent cultures were used for each marker and cell line, except for Th/α-syn colocalization, for which 2 independent cultures were evaluated. The graft survival percentage was calculated from the total transplanted (*n* = 39), showing graft survival via a positive STEM121 signal. This was assessed for each hiPSC line transplanted at the floor-plate stage in sham and 6-OHDA-lesioned SNpc, two mpt. Cell survival was evaluated in at least three brain slides for each rat. To quantify Lmx1a expression, positive cells were counted independently of cellular location; then, we tallied signals within the cell nucleus, outside the nucleus, and in both cellular compartments for every Lmx1a-positive cell using the ImageJ system. Orthogonal cross-sections to analyze Th/STEM121 cellular colocalization were generated using ImageJ’s Orthogonal Views function, with a variable number of slices employed for each image, ranging from 13 to 21. The orthogonal analysis was conducted explicitly in areas where the co-expression of Th and STEM121 exhibited the highest intensity. DAPI was used to normalize the number of cells: all marker counts were conducted by comparison with the number of nuclei stained with DAPI.

### 4.11. Statistical Analysis

All data are presented as the mean ± standard error of the mean (SEM) and 95% confidence interval (CI). Statistical tests and biological replicates are indicated in the figure captions. All statistical analyses were performed with GraphPad Prism v8.0 software for Windows. A one-way ANOVA analysis was carried out to determine statistically significant differences between groups. An alpha level of *p* < 0.01 was set as the threshold for significance. Tukey’s range test was utilized as a post hoc when the ANOVA analysis identified significant differences.

## Data Availability

The datasets used and analyzed during the current study are available from the corresponding author upon reasonable request.

## Supplementary Materials

The following supporting information can be downloaded at https://www.mdpi.com/article/10.3390/life14060728/s1, Figure S1: In vitro characterization of hiPSC lines at day 5 of dopaminergic maturation; Figure S2: Day 15 in vitro characterization of hiPSC lines during dopaminergic maturation; Figure S3. Tyrosine hydroxylase expression in transplanted hiPSC-derived floor-plate progenitors at a low magnification two months post-transplantation; Table S1: Number of turns ipsilateral to the 6-OHDA lesion of rats induced by amphetamine; Table S2: Primary and secondary antibodies used in immunofluorescence.

## References

[1] Lebouvier T., Chaumette T., Paillusson S., Duyckaerts C., Bruley des Varannes S., Neunlist M., Derkinderen P. (2009). The Second Brain and Parkinson’s Disease. Eur. J. Neurosci..

[2] Jankovic J. (2008). Parkinson’s Disease: Clinical Features and Diagnosis. J. Neurol. Neurosurg. Psychiatry.

[3] Poewe W., Seppi K., Tanner C.M., Halliday G.M., Brundin P., Volkmann J., Schrag A.E., Lang A.E. (2017). Parkinson Disease. Nat. Rev. Dis. Primers.

[4] de Lau L.M.L., Schipper C.M.A., Hofman A., Koudstaal P.J., Breteler M.M.B. (2005). Prognosis of Parkinson Disease. Arch. Neurol..

[5] Shulman J.M., De Jager P.L., Feany M.B. (2011). Parkinson’s Disease: Genetics and Pathogenesis. Annu. Rev. Pathol..

[6] Williams-Gray C.H., Mason S.L., Evans J.R., Foltynie T., Brayne C., Robbins T.W., Barker R.A. (2013). The CamPaIGN Study of Parkinson’s Disease: 10-Year Outlook in an Incident Population-Based Cohort. J. Neurol. Neurosurg. Psychiatry.

[7] Ben-Shlomo Y., Darweesh S., Llibre-Guerra J., Marras C., San Luciano M., Tanner C. (2024). The Epidemiology of Parkinson’s Disease. Lancet.

[8] Ou Z., Pan J., Tang S., Duan D., Yu D., Nong H., Wang Z. (2021). Global Trends in the Incidence, Prevalence, and Years Lived With Disability of Parkinson’s Disease in 204 Countries/Territories From 1990 to 2019. Front. Public Health.

[9] Lange L.M., Gonzalez-Latapi P., Rajalingam R., Tijssen M.A.J., Ebrahimi-Fakhari D., Gabbert C., Ganos C., Ghosh R., Kumar K.R., Lang A.E. (2022). Nomenclature of Genetic Movement Disorders: Recommendations of the International Parkinson and Movement Disorder Society Task Force—An Update. Mov. Disord..

[10] Garcia Santa Cruz B., Husch A., Hertel F. (2023). Machine Learning Models for Diagnosis and Prognosis of Parkinson’s Disease Using Brain Imaging: General Overview, Main Challenges, and Future Directions. Front. Aging Neurosci..

[11] Hornykiewicz O., Riederer P., Reichmann H., Youdim M.B.H., Gerlach M. (2006). The Discovery of Dopamine Deficiency in the Parkinsonian Brain. Parkinson’s Disease and Related Disorders.

[12] Porritt M., Stanic D., Finkelstein D., Batchelor P., Lockhart S., Hughes A., Kalnins R., Howells D. (2005). Dopaminergic Innervation of the Human Striatum in Parkinson’s Disease. Mov. Disord..

[13] Spillantini M.G., Schmidt M.L., Lee V.M.-Y., Trojanowski J.Q., Jakes R., Goedert M. (1997). α-Synuclein in Lewy Bodies. Nature.

[14] Braak H., Del Tredici K., Rüb U., de Vos R.A.I., Jansen Steur E.N.H., Braak E. (2003). Staging of Brain Pathology Related to Sporadic Parkinson’s Disease. Neurobiol. Aging.

[15] Burré J., Sharma M., Tsetsenis T., Buchman V., Etherton M.R., Südhof T.C. (2010). α-Synuclein Promotes SNARE-Complex Assembly in Vivo and in Vitro. Science.

[16] Bernal-Conde L.D., Ramos-Acevedo R., Reyes-Hernández M.A., Balbuena-Olvera A.J., Morales-Moreno I.D., Argüero-Sánchez R., Schüle B., Guerra-Crespo M. (2020). Alpha-Synuclein Physiology and Pathology: A Perspective on Cellular Structures and Organelles. Front. Neurosci..

[17] Devi L., Raghavendran V., Prabhu B.M., Avadhani N.G., Anandatheerthavarada H.K. (2008). Mitochondrial Import and Accumulation of α-Synuclein Impair Complex I in Human Dopaminergic Neuronal Cultures and Parkinson Disease Brain. J. Biol. Chem..

[18] Polymeropoulos M.H., Lavedan C., Leroy E., Ide S.E., Dehejia A., Dutra A., Pike B., Root H., Rubenstein J., Boyer R. (1997). Mutation in the α-Synuclein Gene Identified in Families with Parkinson’s Disease. Science.

[19] Singleton A.B., Farrer M., Johnson J., Singleton A., Hague S., Kachergus J., Hulihan M., Peuralinna T., Dutra A., Nussbaum R. (2003). α-Synuclein Locus Triplication Causes Parkinson’s Disease. Science.

[20] Barker R.A., Parmar M., Studer L., Takahashi J. (2017). Human Trials of Stem Cell-Derived Dopamine Neurons for Parkinson’s Disease: Dawn of a New Era. Cell Stem Cell.

[21] Trounson A., DeWitt N.D. (2016). Pluripotent Stem Cells Progressing to the Clinic. Nat. Rev. Mol. Cell Biol..

[22] Björklund A., Parmar M. (2021). Dopamine Cell Therapy: From Cell Replacement to Circuitry Repair. J. Park. Dis..

[23] Devine M.J., Gwinn K., Singleton A., Hardy J. (2011). Parkinson’s Disease and α-Synuclein Expression. Mov. Disord..

[24] Byers B., Cord B., Nguyen H.N., Schüle B., Fenno L., Lee P.C., Deisseroth K., Langston J.W., Pera R.R., Palmer T.D. (2011). SNCA Triplication Parkinson’s Patient’s IPSC-Derived DA Neurons Accumulate α-Synuclein and Are Susceptible to Oxidative Stress. PLoS ONE.

[25] Shrigley S., Nilsson F., Mattsson B., Fiorenzano A., Mudannayake J., Bruzelius A., Ottosson D.R., Björklund A., Hoban D.B., Parmar M. (2021). Grafts Derived from an α-Synuclein Triplication Patient Mediate Functional Recovery but Develop Disease-Associated Pathology in the 6-OHDA Model of Parkinson’s Disease. J. Park. Dis..

[26] Oliveira L.M.A., Falomir-Lockhart L.J., Botelho M.G., Lin K.H., Wales P., Koch J.C., Gerhardt E., Taschenberger H., Outeiro T.F., Lingor P. (2015). Elevated α-Synuclein Caused by SNCA Gene Triplication Impairs Neuronal Differentiation and Maturation in Parkinson’s Patient-Derived Induced Pluripotent Stem Cells. Cell Death Dis..

[27] Dauer W., Kholodilov N., Vila M., Trillat A.-C., Goodchild R., Larsen K.E., Staal R., Tieu K., Schmitz Y., Yuan C.A. (2002). Resistance of α-Synuclein Null Mice to the Parkinsonian Neurotoxin MPTP. Proc. Natl. Acad. Sci. USA.

[28] Chen Y., Dolt K.S., Kriek M., Baker T., Downey P., Drummond N.J., Canham M.A., Natalwala A., Rosser S., Kunath T. (2019). Engineering Synucleinopathy-resistant Human Dopaminergic Neurons by CRISPR-mediated Deletion of the SNCA Gene. Eur. J. Neurosci..

[29] Luk K.C., Kehm V., Carroll J., Zhang B., O’Brien P., Trojanowski J.Q., Lee V.M.-Y. (2012). Pathological Alpha-Synuclein Transmission Initiates Parkinson-like Neurodegeneration in Nontransgenic Mice. Science.

[30] Parmar M., Grealish S., Henchcliffe C. (2020). The Future of Stem Cell Therapies for Parkinson Disease. Nat. Rev. Neurosci..

[31] Grealish S., Diguet E., Kirkeby A., Mattsson B., Heuer A., Bramoulle Y., Van Camp N., Perrier A.L., Hantraye P., Björklund A. (2014). Human ESC-Derived Dopamine Neurons Show Similar Preclinical Efficacy and Potency to Fetal Neurons When Grafted in a Rat Model of Parkinson’s Disease. Cell Stem Cell.

[32] Gaillard A., Decressac M., Frappé I., Fernagut P.O., Prestoz L., Besnard S., Jaber M. (2009). Anatomical and Functional Reconstruction of the Nigrostriatal Pathway by Intranigral Transplants. Neurobiol. Dis..

[33] Thompson L.H., Grealish S., Kirik D., Björklund A. (2009). Reconstruction of the Nigrostriatal Dopamine Pathway in the Adult Mouse Brain. Eur. J. Neurosci..

[34] Wictorin K., Brundin P., Sauer H., Lindvall O., Björklund A. (1992). Long Distance Directed Axonal Growth from Human Dopaminergic Mesencephalic Neuroblasts Implanted along the Nigrostriatal Pathway in 6-Hydroxydopamine Lesioned Adult Rats. J. Comp. Neurol..

[35] Droguerre M., Brot S., Vitrac C., Benoit-Marand M., Belnoue L., Patrigeon M., Lainé A., Béré E., Jaber M., Gaillard A. (2022). Better Outcomes with Intranigral versus Intrastriatal Cell Transplantation: Relevance for Parkinson’s Disease. Cells.

[36] Brot S., Thamrin N.P., Bonnet M.-L., Francheteau M., Patrigeon M., Belnoue L., Gaillard A. (2022). Long-Term Evaluation of Intranigral Transplantation of Human IPSC-Derived Dopamine Neurons in a Parkinson’s Disease Mouse Model. Cells.

[37] Kim T.W., Koo S.Y., Studer L. (2020). Pluripotent Stem Cell Therapies for Parkinson Disease: Present Challenges and Future Opportunities. Front. Cell Dev. Biol..

[38] Maya-Espinosa G., Collazo-Navarrete O., Millán-Aldaco D., Palomero-Rivero M., Guerrero-Flores G., Drucker-Colín R., Covarrubias L., Guerra-Crespo M. (2015). Mouse Embryonic Stem Cell-Derived Cells Reveal Niches That Support Neuronal Differentiation in the Adult Rat Brain. Stem Cells.

[39] Collazo-Navarrete O., Hernández-García D., Guerrero-Flores G., Drucker-Colín R., Guerra-Crespo M., Covarrubias L. (2019). The Substantia Nigra Is Permissive and Gains Inductive Signals When Lesioned for Dopaminergic Differentiation of Embryonic Stem Cells. Stem Cells Dev..

[40] Ramos-Acevedo R., Morato-Torres C.A., Padilla-Godínez F.J., Bernal-Conde L.D., Palomero-Rivero M., Zafar F., Collazo-Navarrete O., Soto-Rojas L.O., Schüle B., Guerra-Crespo M. (2023). Embryoid Body Cells from Human Embryonic Stem Cells Overexpressing Dopaminergic Transcription Factors Survive and Initiate Neurogenesis via Neural Rosettes in the Substantia Nigra. Brain Sci..

[41] Zafar F., Nallur Srinivasaraghavan V., Yang Chen M., Alejandra Morato Torres C., Schüle B. (2022). Isogenic Human SNCA Gene Dosage Induced Pluripotent Stem Cells to Model Parkinson’s Disease. Stem Cell Res..

[42] Brown J.P., Couillard-Després S., Cooper-Kuhn C.M., Winkler J., Aigner L., Kuhn H.G. (2003). Transient Expression of Doublecortin during Adult Neurogenesis. J. Comp. Neurol..

[43] Duly A.M.P., Kao F.C.L., Teo W.S., Kavallaris M. (2022). ΒIII-Tubulin Gene Regulation in Health and Disease. Front. Cell Dev. Biol..

[44] Andersson E., Tryggvason U., Deng Q., Friling S., Alekseenko Z., Robert B., Perlmann T., Ericson J. (2006). Identification of Intrinsic Determinants of Midbrain Dopamine Neurons. Cell.

[45] Gale E., Li M. (2008). Midbrain Dopaminergic Neuron Fate Specification: Of Mice and Embryonic Stem Cells. Mol. Brain.

[46] Kirkeby A., Nolbrant S., Tiklova K., Heuer A., Kee N., Cardoso T., Ottosson D.R., Lelos M.J., Rifes P., Dunnett S.B. (2017). Predictive Markers Guide Differentiation to Improve Graft Outcome in Clinical Translation of HESC-Based Therapy for Parkinson’s Disease. Cell Stem Cell.

[47] Zafar F., Valappil R.A., Kim S., Johansen K.K., Chang A.L.S., Tetrud J.W., Eis P.S., Hatchwell E., Langston J.W., Dickson D.W. (2018). Genetic Fine-Mapping of the Iowan SNCA Gene Triplication in a Patient with Parkinson’s Disease. NPJ Park. Dis..

[48] Zhang J., Jiao J. (2015). Molecular Biomarkers for Embryonic and Adult Neural Stem Cell and Neurogenesis. Biomed. Res. Int..

[49] Chung S., Leung A., Han B.-S., Chang M.-Y., Moon J.-I., Kim C.-H., Hong S., Pruszak J., Isacson O., Kim K.-S. (2009). Wnt1-Lmx1a Forms a Novel Autoregulatory Loop and Controls Midbrain Dopaminergic Differentiation Synergistically with the SHH-FoxA2 Pathway. Cell Stem Cell.

[50] Zetterström R.H., Solomin L., Jansson L., Hoffer B.J., Olson L., Perlmann T. (1997). Dopamine Neuron Agenesis in Nurr1-Deficient Mice. Science.

[51] Maxwell S.L., Ho H.-Y., Kuehner E., Zhao S., Li M. (2005). Pitx3 Regulates Tyrosine Hydroxylase Expression in the Substantia Nigra and Identifies a Subgroup of Mesencephalic Dopaminergic Progenitor Neurons during Mouse Development. Dev. Biol..

[52] Saucedo-Cardenas O., Quintana-Hau J.D., Le W.D., Smidt M.P., Cox J.J., De Mayo F., Burbach J.P., Conneely O.M. (1998). Nurr1 Is Essential for the Induction of the Dopaminergic Phenotype and the Survival of Ventral Mesencephalic Late Dopaminergic Precursor Neurons. Proc. Natl. Acad. Sci. USA.

[53] Abeliovich A., Schmitz Y., Fariñas I., Choi-Lundberg D., Ho W.-H., Castillo P.E., Shinsky N., Verdugo J.M.G., Armanini M., Ryan A. (2000). Mice Lacking α-Synuclein Display Functional Deficits in the Nigrostriatal Dopamine System. Neuron.

[54] Specht C.G., Schoepfer R. (2001). Deletion of the Alpha-Synuclein Locus in a Subpopulation of C57BL/6J Inbred Mice. BMC. Neurosci..

[55] Garcia-Reitboeck P., Anichtchik O., Dalley J.W., Ninkina N., Tofaris G.K., Buchman V.L., Spillantini M.G. (2013). Endogenous Alpha-Synuclein Influences the Number of Dopaminergic Neurons in Mouse Substantia Nigra. Exp. Neurol..

[56] Flierl A., Oliveira L.M.A., Falomir-Lockhart L.J., Mak S.K., Hesley J., Soldner F., Arndt-Jovin D.J., Jaenisch R., Langston J.W., Jovin T.M. (2014). Higher Vulnerability and Stress Sensitivity of Neuronal Precursor Cells Carrying an Alpha-Synuclein Gene Triplication. PLoS ONE.

[57] Mak S.K., Tewari D., Tetrud J.W., Langston J.W., Schüle B. (2011). Mitochondrial Dysfunction in Skin Fibroblasts from a Parkinson’s Disease Patient with an Alpha-Synuclein Triplication. J. Park. Dis..

[58] Chung C.Y., Khurana V., Auluck P.K., Tardiff D.F., Mazzulli J.R., Soldner F., Baru V., Lou Y., Freyzon Y., Cho S. (2013). Identification and Rescue of α-Synuclein Toxicity in Parkinson Patient–Derived Neurons. Science.

[59] Sastre D., Zafar F., Torres C.A.M., Piper D., Kirik D., Sanders L.H., Qi L.S., Schüle B. (2023). Inactive S. *aureus* Cas9 Downregulates Alpha-Synuclein and Reduces MtDNA Damage and Oxidative Stress Levels in Human Stem Cell Model of Parkinson’s Disease. Sci. Rep..

[60] Lin M., Mackie P.M., Shaerzadeh F., Gamble-George J., Miller D.R., Martyniuk C.J., Khoshbouei H. (2021). In Parkinson’s Patient-Derived Dopamine Neurons, the Triplication of α-Synuclein Locus Induces Distinctive Firing Pattern by Impeding D2 Receptor Autoinhibition. Acta Neuropathol. Commun..

[61] Schweitzer J.S., Song B., Herrington T.M., Park T.-Y., Lee N., Ko S., Jeon J., Cha Y., Kim K., Li Q. (2020). Personalized IPSC-Derived Dopamine Progenitor Cells for Parkinson’s Disease. N. Engl. J. Med..

[62] Parmar M., Björklund A. (2020). From Skin to Brain: A Parkinson’s Disease Patient Transplanted with His Own Cells. Cell Stem Cell.

[63] Qiu L., Liao M.-C., Chen A.K., Wei S., Xie S., Reuveny S., Zhou Z.D., Hunziker W., Tan E.K., Oh S.K.W. (2017). Immature Midbrain Dopaminergic Neurons Derived from Floor-Plate Method Improve Cell Transplantation Therapy Efficacy for Parkinson’s Disease. Stem Cells Transl. Med..

[64] Li W., Sun W., Zhang Y., Wei W., Ambasudhan R., Xia P., Talantova M., Lin T., Kim J., Wang X. (2011). Rapid Induction and Long-Term Self-Renewal of Primitive Neural Precursors from Human Embryonic Stem Cells by Small Molecule Inhibitors. Proc. Natl. Acad. Sci. USA.

[65] Qiu X., Liu Y., Xiao X., He J., Zhang H., Li Y. (2019). In Vitro Induction of Human Embryonic Stem Cells into the Midbrain Dopaminergic Neurons and Transplantation in Cynomolgus Monkey. Cell. Reprogram..

[66] Yamada M., Iwatsubo T., Mizuno Y., Mochizuki H. (2004). Overexpression of A-synuclein in Rat Substantia Nigra Results in Loss of Dopaminergic Neurons, Phosphorylation of A-synuclein and Activation of Caspase-9: Resemblance to Pathogenetic Changes in Parkinson’s Disease. J. Neurochem..

[67] Al-Wandi A., Ninkina N., Millership S., Williamson S.J.M., Jones P.A., Buchman V.L. (2010). Absence of α-Synuclein Affects Dopamine Metabolism and Synaptic Markers in the Striatum of Aging Mice. Neurobiol. Aging.

[68] Connor-Robson N., Peters O.M., Millership S., Ninkina N., Buchman V.L. (2016). Combinational Losses of Synucleins Reveal Their Differential Requirements for Compensating Age-Dependent Alterations in Motor Behavior and Dopamine Metabolism. Neurobiol. Aging.

[69] Volpato V., Smith J., Sandor C., Ried J.S., Baud A., Handel A., Newey S.E., Wessely F., Attar M., Whiteley E. (2018). Reproducibility of Molecular Phenotypes after Long-Term Differentiation to Human IPSC-Derived Neurons: A Multi-Site Omics Study. Stem Cell Rep..

[70] Adigbli G., Ménoret S., Cross A.R., Hester J., Issa F., Anegon I. (2020). Humanization of Immunodeficient Animals for the Modeling of Transplantation, Graft Versus Host Disease, and Regenerative Medicine. Transplantation.

[71] Dehmelt L., Halpain S. (2004). The MAP2/Tau Family of Microtubule-Associated Proteins. Genome Biol..

[72] Guerra-Crespo M., Collazo-Navarrete O., Ramos-Acevedo R., Morato-Torres C.A., Schüle B., Turksen K. (2021). Embryoid Body Formation from Mouse and Human Pluripotent Stem Cells for Transplantation to Study Brain Microenvironment and Cellular Differentiation. Embryonic Stem Cell Protocols.

[73] Kilkenny C., Browne W.J., Cuthill I.C., Emerson M., Altman D.G. (2010). Improving Bioscience Research Reporting: The ARRIVE Guidelines for Reporting Animal Research. PLoS Biol..

[74] Deumens R., Blokland A., Prickaerts J. (2002). Modeling Parkinson’s Disease in Rats: An Evaluation of 6-OHDA Lesions of the Nigrostriatal Pathway. Exp. Neurol..

[75] Boronat-García A., Palomero-Rivero M., Guerra-Crespo M., Millán-Aldaco D., Drucker-Colín R. (2016). Intrastriatal Grafting of Chromospheres: Survival and Functional Effects in the 6-OHDA Rat Model of Parkinson’s Disease. PLoS ONE.

[76] Paxinos G., Watson C. (2005). The Rat Brain in Stereotaxic Coordinates.

