# Supplementary material for: Alpha-Synuclein Gene Alterations Modulate Tyrosine Hydroxylase in Human iPSC-Derived Neurons in a Parkinson’s Disease Animal Model
Source: Life (Basel). 2024 Jun 5;14(6):728. doi: 10.3390/life14060728 (PMC11204703; doi:10.3390/life14060728)
Supplement: Supplementary file 1 [file life-14-00728-s001.zip › Bernal-Conde_SupplementaryFigures.pdf]

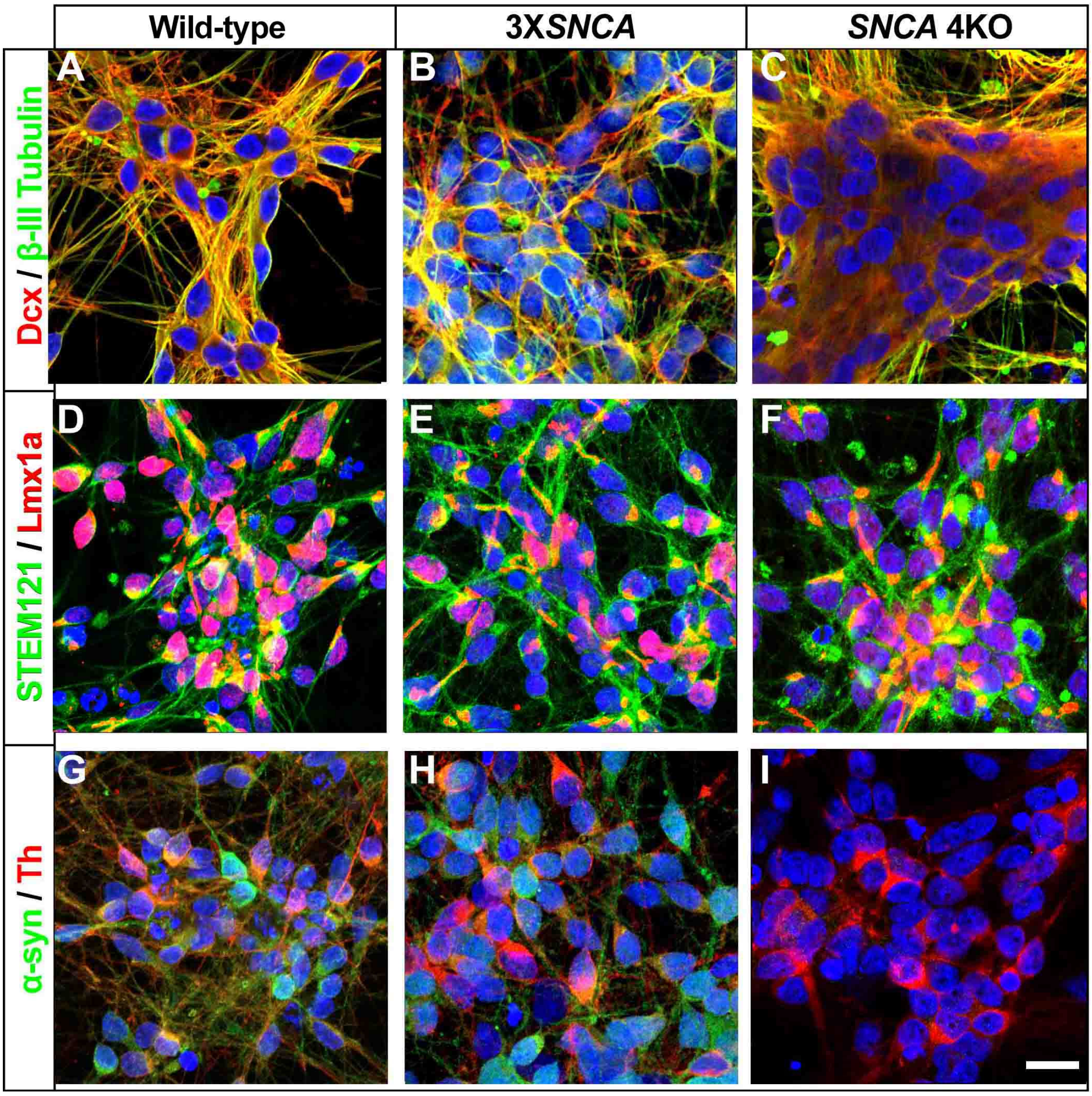

Figure S1. ***In vitro* characterization of hiPSC lines at day 5 of dopaminergic maturation**. Representative photomicrographs of wild-type (A, D, G), 3XSNCA (B, E, H), and SNCA 4KO (C, F, I) hiPSC lines at day 5 of dopaminergic maturation corresponding to the day 30 of cell culture. Images illustrate immunostaining for Dcx/β-III Tubulin (A-C), Lmx1a/STEM121 (D-F), and α-syn/Th (G-I). DAPI (blue) is used for nuclear staining. Scale bar: 15 μm.

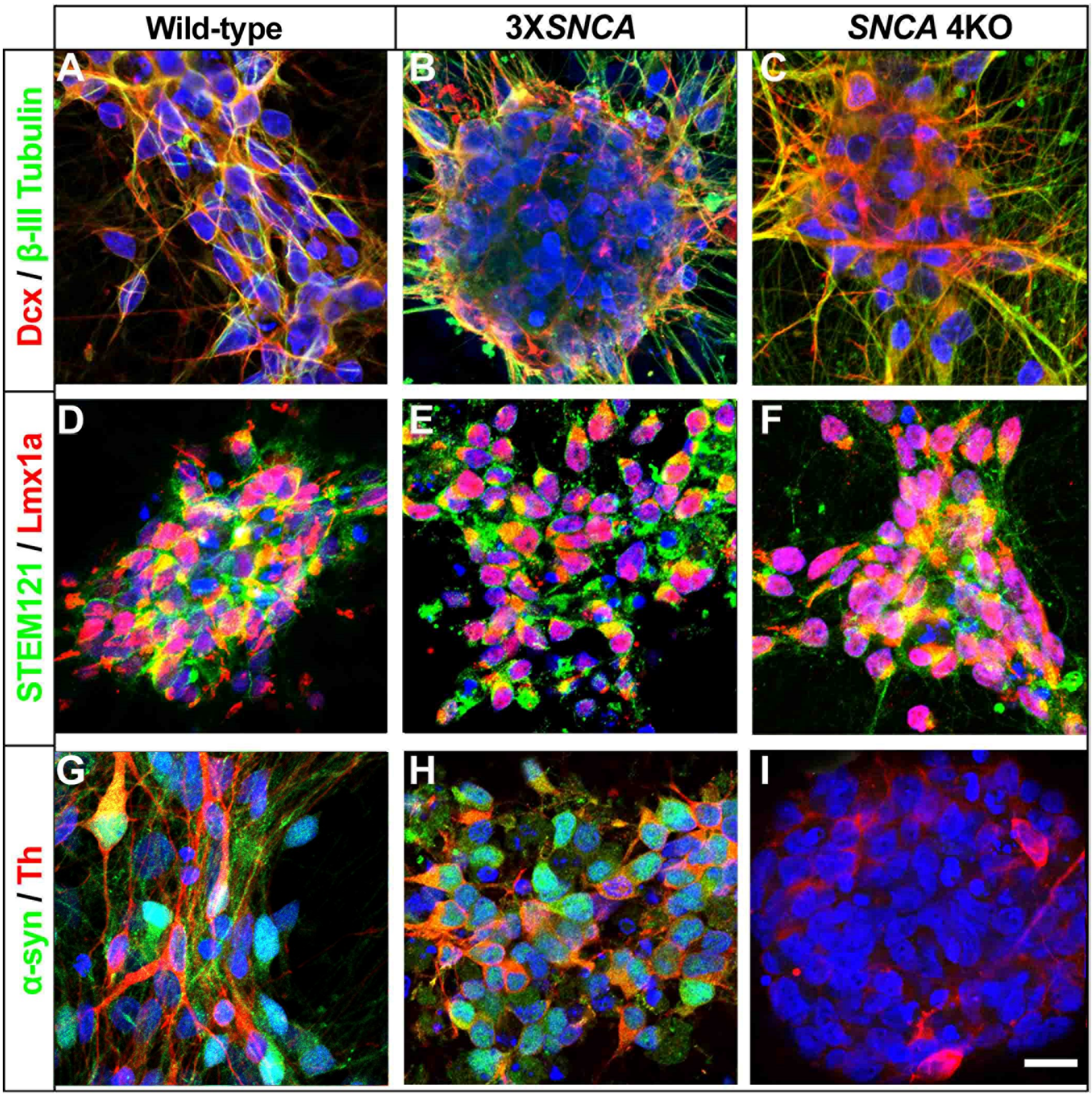

Figure S2. ***In vitro* characterization of hiPSC lines at day 15 of dopaminergic maturation**. Representative photomicrographs of wild-type (A, D, G), 3XSNCA (B, E, H), and SNCA 4KO (C, F, I) hiPSC lines at day 15 of dopaminergic maturation corresponding to the day 40 of cell culture. The images show immunostaining for Dcx/β-III Tubulin (A-C), Lmx1a/STEM121 (D-F), and α-syn/Th (G-I). DAPI (blue) is utilized for nuclear staining. Scale bar: 15 μm.

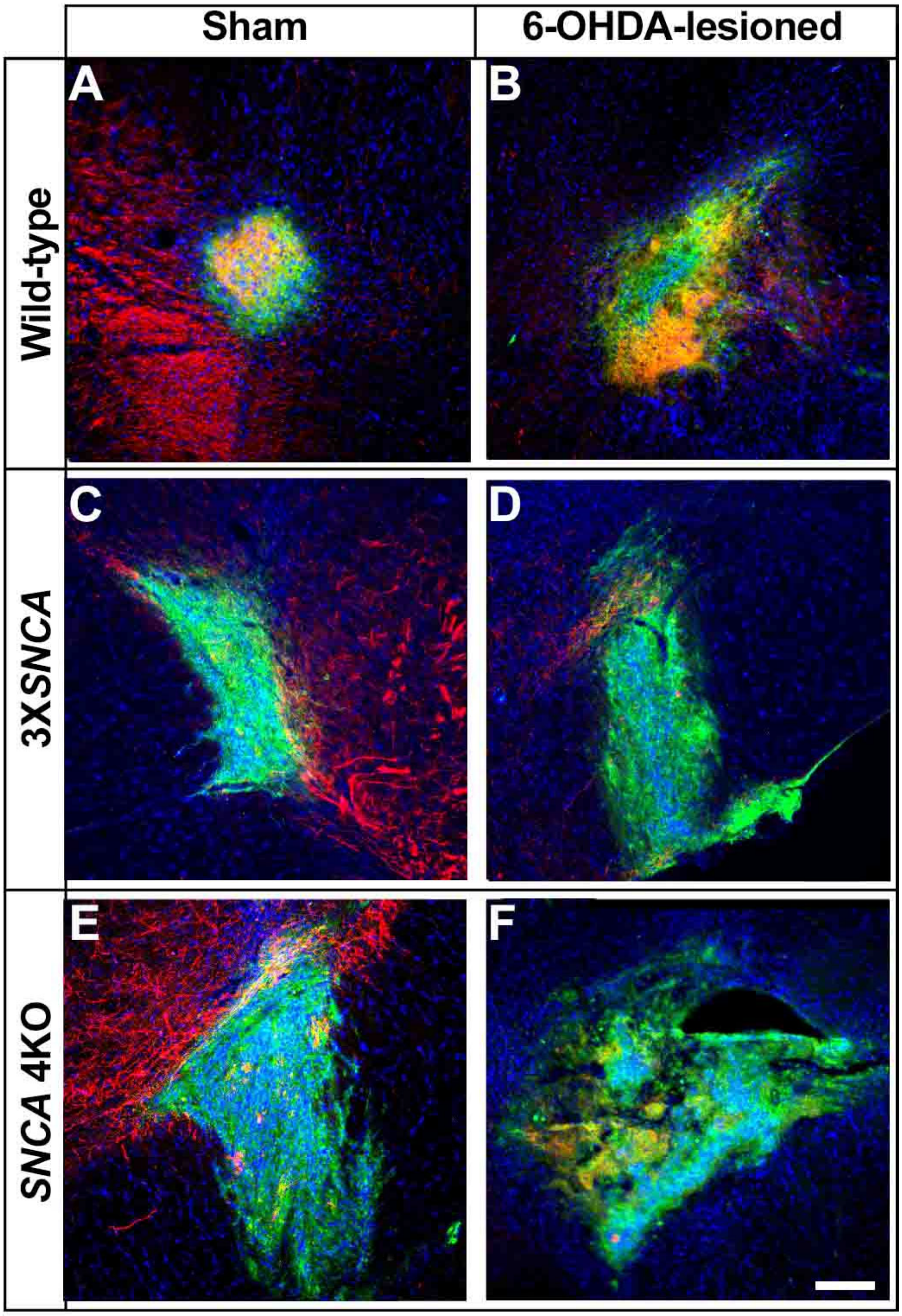

Figure S3. **Tyrosine hydroxylase expression in transplanted hiPSC-derived floor-plate progenitors at a low magnification two months post-transplantation**. Representative photomicrographs of wild-type **(A, B)**, 3X*SNCA* **(C, D)**, and *SNCA* 4KO **(E, F)** hiPSC lines showing Th expression (red) in cells transplanted at the floor-plate phase in the regions where TH-signal was observed, assessed two months post-transplantation. The human cell marker STEM121 (green) was utilized to identify human transplanted cells. DAPI (blue) was used for nuclear staining. Note: the transplants shown are not in Figure 8, except for **(A)**. Scale bar: 150 μm, magnification of 10X.
