# Supplementary material for: Alpha-Synuclein Gene Alterations Modulate Tyrosine Hydroxylase in Human iPSC-Derived Neurons in a Parkinson’s Disease Animal Model
Source: Life (Basel). 2024 Jun 5;14(6):728. doi: 10.3390/life14060728 (PMC11204703; doi:10.3390/life14060728)
Supplement: Supplementary file 1 [file life-14-00728-s001.zip › Bernal-Conde_SupplementaryTableS1.pdf]

Table S1. Number of turns ipsilateral to the 6-OHDA lesion of rats induced by apomorphine.

	**Number of rotations**							
Cell line / Rat #	1	2	3	4	5	6	7	8
Wild-type	425	636	1359	856	889	822	673	744
3X*SNCA*	766	1105	656	466	1039	584	433	408
SNCA 4KO	422	755	1423	1094	968	478	974	615
