# Supplementary material for: Alpha-Synuclein Gene Alterations Modulate Tyrosine Hydroxylase in Human iPSC-Derived Neurons in a Parkinson’s Disease Animal Model
Source: Life (Basel). 2024 Jun 5;14(6):728. doi: 10.3390/life14060728 (PMC11204703; doi:10.3390/life14060728)
Supplement: Supplementary file 1 [file life-14-00728-s001.zip › Bernal-Conde_SupplementaryTableS2.pdf]

Table S2. Primary and secondary antibodies used in immunofluorescence.

**Primary Antibodies**	**Source**	**Host**	**Dilution**
STEM121	Takara	Mouse	1:500
Doublecortin	Santa Cruz Biotechnology	Goat	1:250
β-III Tubulin	Biolegend	Mouse	1:500
Lmx1a	Merck Millipore	Rabbit	1:500
Tyrosine hydroxylase	Merck Millipore	Mouse	1:500
Tyrosine hydroxylase	Merck Millipore	Rabbit	1:500
Alpha-synuclein	Abcam	Rabbit	1:150
Alpha-synuclein	BD biosciences	Mouse	1:500
**Secondary Antibodies**	**Source**	**Host**	**Dilution**
Anti-mouse Alexa Fluor 488	Jackson ImmunoResearch Inc.	Donkey	1:1000
Anti-rabbit Alexa Fluor 594	Jackson ImmunoResearch Inc.	Donkey	1:1000
Anti-goat Alexa Fluor 568	Invitrogen	Donkey	1:400
